# Recurrent Malignant Pericardial Effusion Management: The Pericardio-Peritoneal Window

**DOI:** 10.3390/jcm15010083

**Published:** 2025-12-22

**Authors:** Antonio Mazzella, Giovanni Caffarena, Claudia Bardoni, Giuseppe Nicolosi, Patrick Maisonneuve, Giorgia Cerretani, Giulia Sedda, Luca Bertolaccini, Giorgio Lo Iacono, Monica Casiraghi, Lorenzo Spaggiari

**Affiliations:** 1 Division of Thoracic Surgery, IEO European Institute of Oncology IRCCS, 20141 Milan, Italy; giovanni.caffarena@ieo.it (G.C.); claudia.bardoni@ieo.it (C.B.); giuseppe.nicolosi@unimi.it (G.N.); giorgia.cerretani@ieo.it (G.C.); giulia.sedda@ieo.it (G.S.); luca.bertolaccini@unimi.it (L.B.); giorgio.loiacono@ieo.it (G.L.I.); monica.casiraghi@ieo.it (M.C.); lorenzo.spaggiari@ieo.it (L.S.); 2 Division of Epidemiology and Biostatistics, IEO European Institute of Oncology IRCCS, 20141 Milan, Italy; patrick.maisonneuve@ieo.it; 3 Department of Oncology and Haemato-Oncology, University of Milan, 20141 Milan, Italy

**Keywords:** lung cancer, pericardial effusion, pericardio-peritoneal window, pericardial metastases, malignant pericardial effusion

## Abstract

**Introduction**: Malignant pericardial effusion (MPE) represents a relatively rare complication in various types of solid tumors. Its management is often challenging. One solution can be represented by surgical approaches, including a pericardio-peritoneal window (PPW), which allows draining the fluid into the abdominal cavity. The aim of this study is to investigate the efficacy and long-term outcomes of the PPW procedure as a definitive therapeutic strategy for MPE. **Materials and methods**: We retrospectively and prospectively observed pre-, peri-, and postoperative data of patients undergoing pericardio-peritoneal window creation from 2010 to December 2023 at the European Institute of Oncology (IEO), including the surgical procedures needed, total and specific postoperative complications, 30-day mortality rate, relapse rate, and the treatment of possible relapses. **Results**: A total of 44 consecutive patients underwent a pericardio-peritoneal window. In 28 patients (63.8%) PPW was associated with mono or bilateral videothoracoscopy for pleural biopsies/talc poudrage. In 23 cases, pre-operative percutaneous pericardial drainage (usually 1–2 days before surgery) was performed. No intraoperative deaths were observed. The 30-day mortality was 9% (four patients). We observed pericardial effusion recurrence in three patients at two months and in five patients at six months. In only two cases we treated this condition because of a pre-tamponade condition, treated by percutaneous pericardial drainage. The success rate of the PPW regarding pericardial relapse requiring further procedures was 95.5%. **Conclusions**: Patients presenting with a favorable short-term prognosis benefit from the pericardio-peritoneal window as a safe and effective method for resolving malignant pericardial effusion. Conversely, pericardial drainage is recommended as the most appropriate therapy for those with a less favorable prognosis.

## 1. Introduction

Malignant pericardial effusion (MPE) represents a severe and often late-stage complication in various types of cancer, with lung cancer, breast cancer, and lymphoma being the most frequent primary malignancies [1,2].

This condition arises from the infiltration of the pericardium by cancer cells, leading to fluid accumulation in the pericardial space. The clinical presentation of MPE can range from being asymptomatic to a life-threatening cardiac tamponade, a medical emergency requiring immediate intervention. The presence of MPE is not only a significant cause of morbidity but is also a strong prognostic indicator, often signaling poor prognosis and reduced survival [1,2,3,4].

The management of MPE is challenging, with several therapeutic options available, each with its own advantages and limitations.

A fundamental role, initially, is played by the diagnosis and accurate quantification of a pericardial effusion, whether symptomatic or not. Currently, the diagnostic workup includes rapid-execution examinations such as cardiac ultrasound (echocardiography) and others (MRI or CT scans), which represent the gold standard for evaluation [5]. Non-surgical interventions include pericardiocentesis with or without catheter drainage [4,6,7,8], often followed by intrapericardial sclerosis using agents like talc or tetracycline to induce local fibrosis and prevent recurrence [9,10,11]. While these methods are minimally invasive and can provide rapid symptomatic relief, they are frequently associated with high rates of recurrence and may not be suitable for patients with loculated or thick effusions. Conversely, systemic therapies, such as chemotherapy and radiation therapy, have shown limited efficacy in controlling the effusion itself, primarily because they are not specifically targeted to the pericardial space.

Since the first report of pericardial drainage in 1829, described by Napoleon’s surgeon Larrey [12], the subxiphoid approach has been the most commonly used for the surgical treatment of malignant pericardial effusions [2,3,7]. Although its use for patients who develop tamponade or a pre-tamponade clinical condition remains controversial, the subxiphoid approach guarantees complete opening of the pericardial sac with complete and permanent drainage of the pericardial effusion; it also provides sufficient histologic, cytologic, and microbiologic material for diagnostic study [2,7].

For a more definitive and long-lasting solution, surgical approaches are often considered. These include the creation of a pericardial window—either into the pleural cavity (pleuro-pericardial window) or, less commonly, into the peritoneal cavity (pericardio-peritoneal window—PPW). The primary goal of these procedures is to create a permanent drainage pathway for the fluid. The pericardio-peritoneal window, by draining the fluid into the large capacity of the abdominal cavity, represents a potentially advantageous alternative for selected patients. However, the long-term efficacy and safety profile of this specific procedure, particularly when performed using minimally invasive techniques, requires further investigation.

The aim of this study is to investigate the efficacy and long-term outcomes of the PPW procedure as a definitive therapeutic strategy for MPE. We hypothesize that this surgical approach provides superior and more durable relief from MPE compared to other palliative methods, ultimately improving the quality of life and the potential survival for a select patient population. By conducting a comprehensive analysis of pre-, intra-, and postoperative outcomes, we seek to provide new insights into the role of this specific surgical modality and its potential to improve the management of and outcomes for patients with MPE.

## 2. Materials and Methods

We retrospectively (until December 2021) and prospectively (from January 2022 to December 2023) observed pre-, peri-, and postoperative data of patients undergoing pericardio-peritoneal window creation from 2010 to December 2023 at the European Institute of Oncology (IEO) in order to definitively treat malignant pericardial effusion resulting from metastasis linked to other cancers.

The patients’ medical and operative records were reviewed in terms of original intervention, time between the first surgery and possible relapse, postoperative comorbidities, and mortality after pericardio-peritoneal window creation and consequent follow-up. The study was conducted in accordance with the Declaration of Helsinki and is reported in accordance with the STROBE (STrengthening the Reporting of OBservational studies in Epidemiology) guidelines. The study was approved by the Ethics Committee of the European Institute of Oncology (UID 3516, 03/12/2021). Written informed consent to undergo the procedure and to use clinical and imaging data for scientific or educational purposes, or both, was obtained from all patients before the operation. Data were collected on demographic outcomes, preoperative ultrasound and computed tomography (CT) findings, anatomical and histological outcomes of the neoplasm, and intraoperative or additional surgical procedures needed. In addition, we investigated total and specific postoperative complications, 30-day mortality rate, hospital stay, relapse rate, treatment of any relapses, and, more generally, OS.

### 2.1. Pre-Operative Management

**Figure 1:**
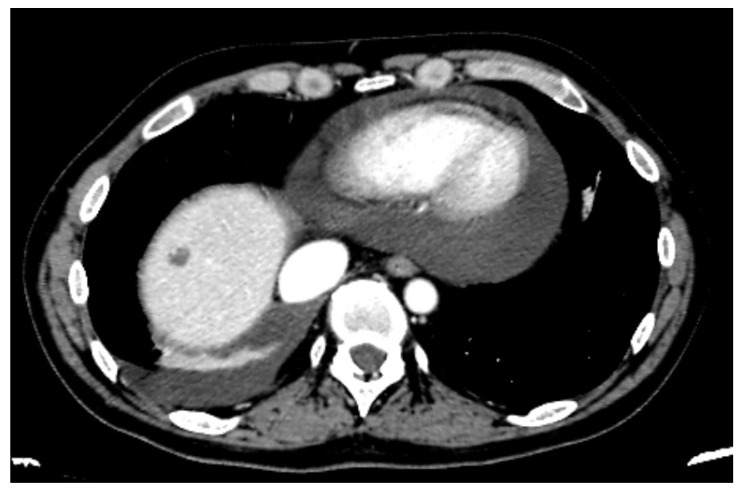
Preoperative CT scan revealing pericardial and right pleural effusion.

Pre-operative staging consisted of a total body computed tomography scan (CT scan) and a positron emission tomography (PET) with fluorodeoxyglucose (FDG). In these cases, a cardiological and pulmonary evaluation is mandatory, particularly an echocardiogram. This exam, compared to the CT scan, is always performed (for the suspicion or diagnosis of pericardial effusion) to evaluate the effective fluid quantity and to assess heart function; it highlights diastolic right ventricular collapse, which is the widely accepted hallmark sign of cardiac tamponade [5,13]. This allows for a more accurate estimation and quantification of the maximum detachment between the pericardium and the cardiac surface [14] than a CT scan, but it is also able to indicate qualitative references of liquid (organized, loculated, or hematic) (Figure 1).

### 2.2. Pericardial–Peritoneal Window: When?

It is crucial to identify the correct indications for the creation of a pericardial window. It is necessary to consider that these patients are affected by stage IV neoplasm, often with multiple metastatic localizations, where the pericardium is only one of the metastatic sites. Therefore, this intervention, sometimes associated with VATS for talc poudrage, has a diagnostic (via pericardial biopsies) and palliative (definitive control and avoidance of pericardial effusion) utility. For this reason, patient selection is very severe, within a multidisciplinary approach, in close agreement with oncologists. The preoperative clinical conditions represent a “conditio sine qua non” for candidacy for surgical intervention. An ECOG score ≥ 3, poor general condition, or extensive metastatic disease represent potential contraindications that may favor simple pericardial drainage, considering the potential complications associated with surgical intervention. Secondly, oncological prognosis (>6 months)—the failure of prior treatments, such as pericardiocentesis or pericardial drainage, which constitute the initial up-front management performed by cardiologists or interventional radiologists—is another criterion. Finally, the confirmed involvement of the pericardium and the correct preoperative quantification of the effusion are required (pre-tamponade or tamponade). In this case, we always prefer draining the pericardial space before surgery (1–2 days before), according to our interventional cardiologists/radiologists, in order to prevent paradoxical hemodynamic instability.

Previous cardiac, hepatic, or abdominal surgeries could represent relative contraindications, which may be linked to previous surgical adhesions that could complicate the patency of the window.

We considered recurrence to be the presence of pericardial effusion > 400 mL, determining a relevant clinical impact with pre-tamponade or tamponade conditions. Every patient underwent monthly cardiologic follow-up with a scheduled echocardiogram.

### 2.3. Surgical Technique

**Figure 2:**
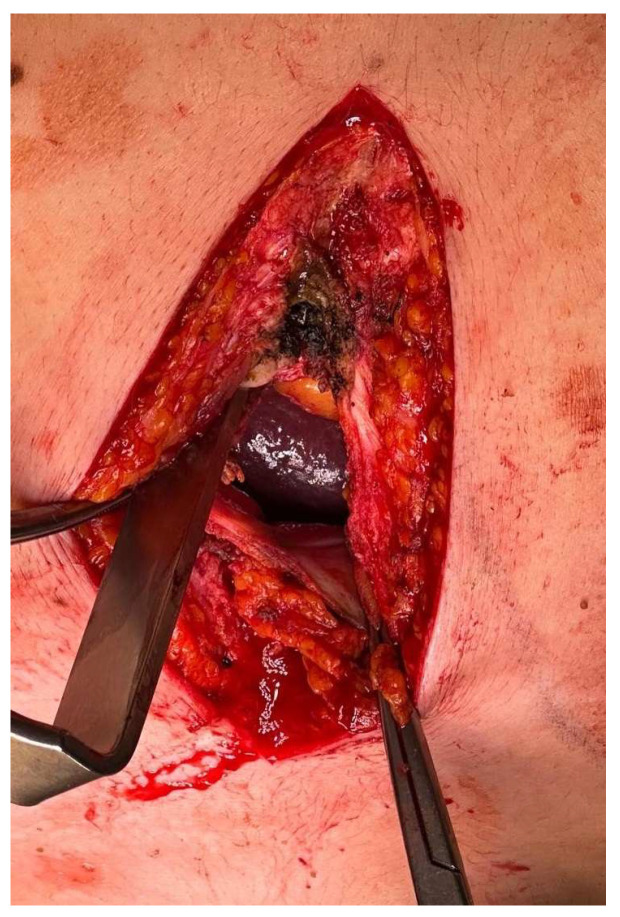
Creation of pericardial–peritoneal window: longitudinal incision performed at the level of the xiphoid process. The figure shows the xiphoid process on top and, in a deeper plane, the pericardial fat, the heart, and the opened pericardial sac. At the bottom, the figure shows the liver with the opened peritoneal sac and diaphragm, in order to establish a transdiaphragmatic communication (or fenestration) between the cavities.

Pericardial–peritoneal window creation, already described in our previous report [2], was performed under general anesthesia with single-lumen orotracheal intubation and mechanical ventilation. A subxiphoid longitudinal approach was performed in order to gain access to the xiphoid process (which can also be resected if operative field exposure is difficult) and the upper abdomen. A longitudinal incision along the pericardial anteroinferior surface and the apical part of the peritoneum was performed. The central and retrosternal portion of the diaphragm was then incised to establish a transdiaphragmatic communication between the pericardial and peritoneal cavities (Figure 2). A pericardial biopsy was always performed in order to obtain a definitive histological diagnosis. When the investigation of the pericardial sac was complex due to pericardial adhesions, a video mediastinoscope was used to explore the pericardial sac or to guide any biopsies.

Then, the margins of the opened pericardium, diaphragm, and peritoneum were subsequently approximated and secured with interrupted non-absorbable sutures on both sides, ensuring the patency of the newly created tract. This suture, in some cases, can also be achieved using mechanical staplers, which allow for a direct connection of the layers, or using energy devices (Ligasure) reinforced by non-absorbable sutures. Finally, an intrapericardial drain was placed and typically removed 24 h post-procedure following confirmation of the absence of significant bleeding or major effusion.

### 2.4. Statistical Methods

Summary statistics of clinicopathological characteristics of patients and age at the date of surgery were produced and tabulated as counts and either percentage or mean, standard deviation (SD), and min and max for categorical and continuous variables, respectively. OS was defined as the time elapsed from surgery to the patient’s death and estimated by the Kaplan–Meier method.

Postoperative death was defined as 30-day mortality or longer if mortality occurred during hospitalization. Survival time for patients still alive at the last follow-up date was considered censored.

Cumulative incidence of relapse after intervention was estimated using the cumulative incidence function with death considered as a competing risk event. Patients who were still alive were censored at the date of the last follow-up. Overall survival was plotted using the Kaplan–Meier method. All statistical analyses were performed using SAS software version 9.4 (Cary, NC, USA).

## 3. Results

From 2010 to 2023, 44 consecutive patients underwent pericardio-peritoneal window creation to treat malignant pericardial effusion (Table 1 and Table 2). Twenty-two patients were male, and twenty-two were female; the mean age was 63 years (range, 41–81 years). Pericardial effusion cytology was positive in 38 (86%) cases and negative in 6 (14%). A total of 36 patients had lung cancer, 5 had breast ductal carcinomas, 1 papillary serous ovarian adenocarcinoma, 1 renal cell carcinoma, and 1 malignant mesothelioma. In six cases, operative pericardial histologic exams revealed non-neoplastic cells but only fibrotic or inflammatory tissue. Among the 36 patients with lung cancer, 33 had adenocarcinoma, 1 had adenosquamous carcinoma, 1 had sarcomatoid, and 1 had non-small-cell lung cancer not otherwise specified.

Pre/tamponade or tamponade condition with an initial hemodynamic instability was diagnosed in five and two cases, respectively. A total of 15 patients (34%) underwent only the pericardio-peritoneal window procedure. In one case (2.2%), the pericardial window was associated with only a chest drain. In the other 28 cases (63.8%), PPW was associated with right VATS (21 cases), left VATS (4 cases), or bilateral VATS (3 cases) for pleural biopsies and talc poudrage.

The suture of the pericardial/diaphragmatic/peritoneum margins was assured by non-absorbable suture in 32 patients (72%). In two patients (11.3%), the window packaging was assured by a stapler (Endo GIA™, purple recharge); in the other seven (16%) patients, the margins were sealed by an energy device (Ligasure™, Covidien) and reinforced by non-absorbable suture in two of these patients.

In 23 cases, preoperative percutaneous pericardial drainage (usually 1–2 days before surgery) was performed in order to stabilize the patient’s hemodynamic aspect in view of the surgery.

Intraoperative mean volume of the drained pericardial effusion was 550 mL (range, 200–1600 mL); mean duration of the procedure was 91.1 min (range, 37–145 min); postoperative mean length of stay was 4.4 days (range, 2–7 days); and eight (18.1%) patients had postoperative complications: six (13.6%) had atrial fibrillation (Minor, Grade II), two (4.5%) had acute respiratory failure requiring ICU admission and non-invasive ventilation (Grade IIIa), one (2.2%) had bilateral pulmonary embolism (Grade II), and one (2.2%) had acute renal injury (Grade 2).

No intraoperative deaths were observed. The 30-day mortality was 9% (four patients). One of them died 6 h after the procedure for an acute cardio-respiratory insufficiency in the ICU. The other three patients died from respiratory failure (two) and multiorgan failure (one).

**Figure 3:**
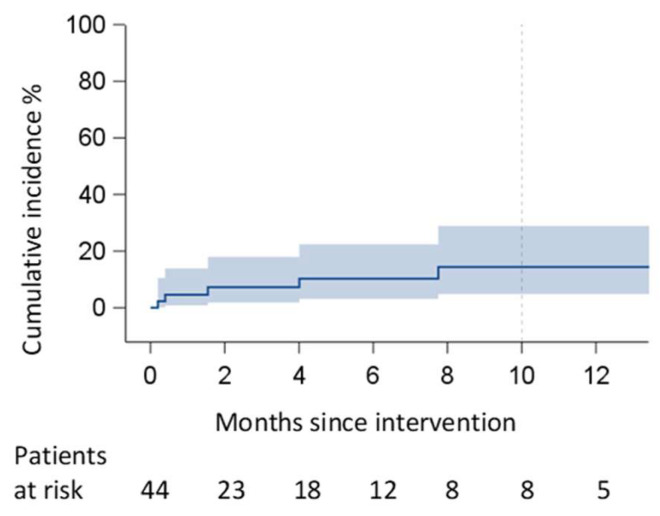
Cumulative incidence of relapse.

**Figure 4:**
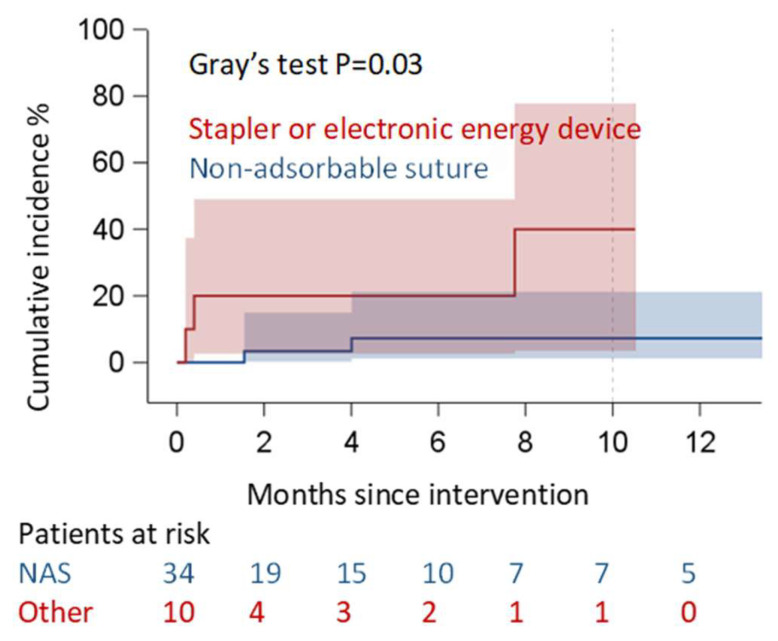
Cumulative incidence of recurrence according to type of suture for PPW.

We observed pericardial effusion recurrence in five patients (three within two months, two more patients within six months) at six months (Figure 3). In three of them, we observed a non-clinically relevant, organized, and loculated effusion. In the other two cases (one within two and the other one within six months), we detected a pre-tamponade condition, which was treated by percutaneous pericardial drainage. Thus, the success rate of the PPW regarding pericardial relapse within 6 months was 88.7%, but if we consider the necessity of further interventions, the success rate increased to 95.5% (2 cases out of 44). Indeed, in the other three cases out of five, we only set up an echocardiographic follow-up because of the absence of signs of heart failure. Analyzing the main risk factors, the statistical analysis did not reveal any technical or demographic factors associated with recurrence. The only statistical significance was reached by the surgical technique for creating the pleuropericardial window (Table 2, Figure 4). Indeed, the use of energy devices, compared to the use of non-absorbable sutures, appears to be associated with a greater possibility of effusion recurrence (*p* = 0.009 (Table 2)).

Median follow-up was 7.19 months. The 6-month overall survival rate was 56.8% (25 patients). Among these patients, only one patient (4%) had pericardial effusion relapse (after one month), which was treated by percutaneous pericardial drainage. Among the 17 patients (43.2%) who died within 6 months after surgery, 13 (76.4%) of them died due to oncological disease progression.

## 4. Discussion

The buildup of fluid around the heart (pericardial effusion) is a relatively common outcome in patients with neoplastic diseases, posing a severe life-threating condition. This threat is linked both to the poor prognosis and to the cardiological implications of a cardiac pre-tamponade or tamponade [15]. With the advent of new therapies (immunotherapy, biological therapies, and specific hormonal therapies), this condition is decreasing. However, when it does occur, it must be addressed as quickly as possible to ensure the patient’s best possible quality of life and to optimize oncological treatment [2,3,4,15].

The optimal approach to metastatic pericardial effusion should both promptly alleviate cardiac tamponade and effectively prevent relapse. Pericardiocentesis by itself is linked to a significant recurrence rate (60–100%), even when performed multiple times [4,6]. On the other hand, pericardial drainage with or without a sclerosing agent injection may optimize the management of these patients, but its efficacy is variable [4,9,10]. A surgical approach (via thoracotomy, VATS, or subxiphoid access), consisting of creating a pericardial window with the pleura or the peritoneum, represents the best option, with satisfactory medium- and long-term results in resolving pericardial effusion.

In this context, these methods ensure definitive control of any recurrence of the effusion; secondly, they also allow for the performance of pleural or pericardial biopsies for a correct histological typing [4,16,17,18].

The success of a pleuro-pericardial window, even if easier to perform via videothoracoscopy, can be affected by several factors. Indeed, the fenestration may be obliterated due to fibrotic events or adhesions linked to lung reventilation or, even worse, to pleural talc poudrage. These conditions would subsequently cancel out the efficacy and efficiency of the window. Illustrating this point, one of our patients, who underwent a pleuro-pericardial window less than 30 days earlier, subsequently required urgent creation of a pericardio-peritoneal window due to the early recurrence of pericardial effusion.

In contrast, the creation of a pericardio-peritoneal window (PPW) is perhaps the most effective technique for the safe and permanent management of recurrent pericardial effusion.

The initial benefit is derived from the creation of communication between the caudal pericardial sac and the cranial peritoneum, enabling continuous, gravity-dependent drainage of the pericardial fluid into the abdominal cavity. Fluid absorption is inherently more efficient within the peritoneal space, eliminating the risk of cardiological sequelae [2,16]. A second advantage is the significantly reduced likelihood of window occlusion, which is attributed to the anatomical disposition of the abdominal organs.

A further advantage lies in the fact that, in cases of organized or loculated effusions, the subxiphoid approach provides the capability to insert a videomediastinoscope or to perform a finger or gentle dissection, therefore resolving any intrapericardial flocculations or organized effusions.

Conversely, however, this procedure requires opening the peritoneal cavity and is clearly more technically demanding than the creation of a simple pleuro-pericardial window. It is, in this context, contraindicated in cases of prior abdominal or hepatic surgery.

Regarding the surgical procedure’s safety and its impact on these patients, no intraoperative mortality was observed in our patient population. The 30-day mortality rate was 9%, attributed to heart failure in one case or complications related to the patient’s respiratory status in the other three cases.

Considering the general condition of these patients at the time of surgery, the 30-day morbidity outcomes (18%—8 out of 44 patients) are also considered entirely acceptable, including minor complications as well as atrial fibrillation (six patients) or bilateral pulmonary embolism treated and resolved with anticoagulant therapy (two cases). We observed no abdominal or “strictly surgical” complications, and 30-day mortality cases were linked to heart failure or ARDS/multiorgan failure, probably linked to oncologic pathology. The morbidity/mortality risk, related to the PPW (which is safe in this well-defined population), is linked more to the surgical intervention with associated anesthesiological implications, rather than to the surgical procedure itself.

We did not find any risk factors associated with the incidence of relapse in our population, with the exception of the use of energy devices to create the PPW. Regarding this aspect, this could be related to the creation of a “tunnel” joining the layers of pericardium, peritoneum, and diaphragm with an energy device. The use of the latter, compared to the use of separate non-absorbable sutures, may perhaps promote the development of postoperative adhesions that might reduce the caliber or even completely obliterate the window. This result should be interpreted with caution, given the small sample size and the limited number of events in these subgroups, which prevents drawing absolute conclusions.

A pivotal aspect of the success rate of this procedure is certainly the correct selection of patients, encompassing oncological status as well as clinical and cardiological parameters.

The first aspect concerns the patient’s oncological prognosis. It is quite evident that a patient whose life expectancy exceeds six months should be considered eligible for this procedure. Conversely, if the prognosis is poorer, less invasive treatments such as simple pericardiocentesis or pericardial drainage would be preferable.

In conjunction with this aspect, the patient’s general condition—which is also closely linked to the oncological prognosis—is of paramount importance.

Thirdly, the accurate instrumental quantification of the effusion and its hemodynamic implications is mandatory. This assessment, performed in close cooperation with cardiologists, radiologists, and interventional cardiologists, is essential, not only in patient selection but also, specifically, in correctly planning all procedural steps leading to the creation of a pericardio-peritoneal window [16,17,18,19]. Undeniably, a condition of pre-tamponade or tamponade is an indication for a pericardio-peritoneal window. In these specific cases, however, the potential association with pleural effusions drives our choice to drain the pericardium (pericardiocentesis or CT/echo-guided pericardial drainage) one or two days before surgery. This practice helps prevent additional cardiac and hemodynamic stress on the patient during anesthesia induction or paradoxical hemodynamic instability [20]. In our series preoperative percutaneous pericardial drainage was performed in 23 cases.

Lastly, failure of any other less invasive attempts to resolve the issue is another potential indication for PPW.

In our population the success rate of PPW regarding pericardial relapse requiring a further medical or surgical intervention was 95.5%. Indeed, we observed a pericardial effusion recidivism in five patients; however, in three of them, no pharmacological or surgical approaches were adopted because of a non-clinically relevant, organized, and loculated effusion. In only two cases (4.5%), we observed a pre-tamponade condition, which was treated by percutaneous pericardial drainage (one case) or reintervention (one case). We did not emphasize or thoroughly explore topics such as overall survival in this discussion for several reasons. Primarily, OS does not represent the scope of this work, which is fundamentally based on the feasibility and efficacy of palliative treatment for pericardial effusions. Furthermore, this cohort consists of a heterogeneous group of patients presenting with metastatic disease, including different NSCLC histotypes, as well as breast, gastrointestinal, and other malignancies. However, even if 6-month overall survival was 56.8% (25 patients), the key takeaway is that only one of these patients had pericardial effusion relapse (after one month), and no patients had hemodynamic issues after the PPW, with complete resolution of pericardial effusions during oncological treatments after diagnosis.

Our study presents several limitations. Firstly, the retrospective nature of our evaluation spans a relatively long period during which oncologic management has significantly evolved. Secondly, the study involves several malignancies, whose aggressiveness and evolution are distinctly different. Thirdly, the limited sample size represents another limitation, which is, nevertheless, inherent to the rarity of the pathology and the specific surgical indications.

Another limitation in our experience is the impossibility of comparing the PPW with other techniques (such as pleuropericardial windows), as they are not part of our common practice.

## 5. Conclusions

Patients presenting with a favorable short-term prognosis could benefit from the pericardio-peritoneal window as a safe and effective method for resolving malignant pericardial effusion. Particularly it may be considered for patients with expected survival >6 months and adequate performance status; less invasive drainage is reasonable for those with a limited prognosis.

## Figures and Tables

**Table 1:** Continuous variables of the population.

Variable	N	Mean +/− SD	Min–Max
Age at surgery	44	62.7 +/− 11.9	41–84
Drained pericardial effusion volume (mL)	36	446 +/− 332	100–600
Ejection fraction (%)	32	58 +/− 7.5	28–70
Duration of surgery (min)	44	91 +/− 33.8	37–155
Length of hospital stay (days)	43	4 +/− 1.01	2–5

**Table 2:** Clinicopathological outcomes (categorical variables) of the population. PPW: pericardial-peritoneal window. ICU: intensive care unit.

Variable	N (%)	Relapse	*p*-Value Gray Test
**Sex** - Male - Female	22 (50.0%) 22 (50.0%)	4 1	0.13
**Primary neoplasm** - Lung - Breast cancer - Ovarian cancer - Renal cancer - Mesothelioma	36 (81.8%) 5 (11.4%) 1 (2.3%) 1 (2.3%) 1 (2.3%)	5 0 0 0 0	0.90
**ECOG** - 0 - 1 - 2 - >2	5 (11.3%) 20 (45.5%) 16 (36.6%) 3 (6.8%)		
**Effusion cytology** - Negative - Positive	6 (13.7%) 38 (86.3%)		NS
**Type of surgery** - PPW alone - PPW + monolateral VATS - PPW + bilateral VATS - PPW + chest drain	15 (34.1%) 25 (56.8%) 3 (6.8%) 1 (2.3%)	2 3 0 0	0.94
**Type of suture for PPW** - Non-adsorbable suture - Stapler - Electronic energy device - Electronic energy device + non-adsorbable suture	32 (72,7%) 5 (11.4%) 5 (11.4%) 2 (4.5%)	2 2 1 0	0.009
**Postoperative complications** - Atrial fibrillation - Acute respiratory failure necessitating ICU stay - Pulmonary embolism - Acute renal injury	4 (9.1%) 2 (4.5%) 1 (2.2%) 1 (2.2%)		
**Relapses at six months** - Yes (non-clinically relevant, only follow-up) - Yes, treated by pericardial drainage - No	3 (6.8%) 2 (4.5%) 39 (88.7%)		

## Data Availability

The original contributions presented in this study are included in the article. Further inquiries can be directed to the corresponding author.
